# Development of a New DNA Marker for Fusarium Yellows Resistance in *Brassica rapa* Vegetables

**DOI:** 10.3390/plants10061082

**Published:** 2021-05-27

**Authors:** Naomi Miyaji, Mst Arjina Akter, Chizuko Suzukamo, Hasan Mehraj, Tomoe Shindo, Takeru Itabashi, Keiichi Okazaki, Motoki Shimizu, Makoto Kaji, Masahiko Katsumata, Elizabeth S. Dennis, Ryo Fujimoto

**Affiliations:** 1 Graduate School of Agricultural Science, Kobe University, Rokkodai, Nada-ku, Kobe 657-8501, Japan; 189a362a@stu.kobe-u.ac.jp (M.A.A.); hmehraj34@stu.kobe-u.ac.jp (H.M.); 2 Iwate Biotechnology Research Center, Narita, Kitakami, Iwate 024-0003, Japan; m-shimizu@ibrc.or.jp; 3 Department of Plant Pathology, Bangladesh Agricultural University, Mymensingh 2202, Bangladesh; 4 Watanabe seed Co., Ltd., Machiyashiki, Misato-cho, Miyagi 987-0003, Japan; ws-ken@chive.ocn.ne.jp (C.S.); ws-kaji@chive.ocn.ne.jp (M.K.); ws-katsu@aroma.ocn.ne.jp (M.K.); 5 Miyagi Prefectural Agriculture and Horticulture Research Center, Natori, Miyagi 981-1243, Japan; shindo-to469@pref.miyagi.lg.jp (T.S.); itabashi-ta714@pref.miyagi.lg.jp (T.I.); 6 Graduate School of Science and Technology, Niigata University, Ikarashi, Nishi-ku, Niigata 950-2181, Japan; okazaki@agr.niigata-u.ac.jp; 7 CSIRO Agriculture and Food, Canberra, ACT 2601, Australia; Liz.Dennis@csiro.au; 8 School of Life Science, Faculty of Science, University of Technology Sydney, P.O. Box 123, Broadway, NSW 2007, Australia

**Keywords:** Fusarium yellows, *Fusarium oxysporum* f. sp. *rapae*, DNA marker, *R* gene, marker-assisted selection, QTL-seq, *Brassica rapa*

## Abstract

In vegetables of *Brassica rapa* L., *Fusarium oxysporum* f. sp. *rapae* (*For*) or *F. oxysporum* f. sp. *conglutinans* (*Foc*) cause Fusarium yellows. A resistance gene against *Foc* (*FocBr1*) has been identified, and deletion of this gene results in susceptibility (*focbr1-1*). In contrast, a resistance gene against *For* has not been identified. Inoculation tests showed that lines resistant to *Foc* were also resistant to *For*, and lines susceptible to *Foc* were susceptible to *For*. However, prediction of disease resistance by a dominant DNA marker on *FocBr1* (Bra012688m) was not associated with disease resistance of *For* in some komatsuna lines using an inoculation test. QTL-seq using four F_2_ populations derived from *For* susceptible and resistant lines showed one causative locus on chromosome A03, which covers *FocBr1*. Comparison of the amino acid sequence of *FocBr1* between susceptible and resistant alleles (*FocBr1* and *FocBo1*) showed that six amino acid differences were specific to susceptible lines. The presence and absence of *FocBr1* is consistent with *For* resistance in F_2_ populations. These results indicate that *FocBr1* is essential for *For* resistance, and changed amino acid sequences result in susceptibility to *For*. This susceptible allele is termed *focbr1-2*, and a new DNA marker (focbr1-2m) for detection of the *focbr1-2* allele was developed.

## 1. Introduction

*Brassica rapa* L. comprises a variety of vegetables that are rich sources of nutrients including vitamins, minerals, dietary fiber, and phytochemicals [1,2]. In leafy vegetables of *B. rapa*, there are two morphotypes, heading types such as Chinese cabbage (var. *pekinensis*) and non-heading type such as pak choi (var. *chinensis*), komatsuna (var. *perviridis*) or chijimina (var. *narinosa*). Root vegetables such as turnip (var. *rapa*) also belong to *B. rapa* [1,2]. Most commercial cultivars of these vegetables are F_1_ hybrids, and hybrid vigor, disease resistance, and late bolting are important breeding traits [3,4,5]. In particular, disease resistance is demanded by farmers, especially for soil-borne diseases that are difficult to control with chemicals [5,6].

Plants have evolved their immunity to pathogens via two mechanisms [7,8]. Pattern recognition receptors (PRRs) located in the plant cell membrane recognize pathogen-associated molecular patterns (PAMPs), which activate PAMP-triggered immunity (PTI) and restrict pathogen development. Most pathogens secrete effectors (avirulence (AVR) proteins) into plant cells to suppress PTI, while plants have various *resistance* (*R*) genes, which mainly encode Toll/Interleukin-1 receptor (TIR) or coiled-coil (CC), nucleotide-binding site (NBS), and leucine-rich repeat (LRR) domains, to detect effectors. Recognition of effectors by R proteins induces effector-triggered immunity (ETI), and recognition of specific effectors by R proteins is termed “gene-for-gene resistance” [7].

*Fusarium oxysporum* is a soil-borne fungus and comprises 150 host-specific formae speciales. *F. oxysporum* causes yellows in a wide range of host plants [5,6]. In *B. rapa* vegetables, two formae speciales of *F. oxysporum* f. sp. *conglutinans* (*Foc*) and f. sp. *rapae* (*For*) have been identified as causing Fusarium yellows [9]. *Foc* was first reported as a causal agent of yellowing in cabbage (*Brassica oleracea* L. var. *capitata*) in 1913 [5,6] and causes Fusarium yellows not only in *B. oleracea* vegetables including cabbage or broccoli but also in *B. rapa* vegetables including turnip, komatsuna, and pak choi [9,10,11,12,13]. In contrast, *For* causes yellowing in *B. rapa* vegetables, but not in *B. oleracea* vegetables [9].

The Fusarium yellows *R* gene against *Foc* has been identified in *B. rapa* (*FocBr1*) and *B. oleracea* (*FocBo1*) [11,12,13,14]. *FocBr1* and *FocBo1* are orthologs and encode a TIR-NBS-LRR protein. In *B. rapa*, an approximately 35-kb deletion including *FocBr1* results in susceptibility (*focbr1-1*) and there are no reports of other causative mutations for susceptibility [12,15]. In contrast, there are three different susceptible alleles of *FocBo1* in *B. oleracea* (*focbo1-1*, *focbo1-2* and *focbo1-3*), but a 35-kb deletion similar to that in Fusarium yellows susceptible lines of *B. rapa* has not been identified [11,13,16,17].

The Fusarium yellows *R* gene against *For* (*ForBr1*) has not been identified. In this study, we performed QTL-seq to isolate *ForBr1*. We developed a DNA marker that can identify the susceptible alleles of Fusarium yellows and tested this marker in cultivars of *B. rapa* vegetables.

## 2. Results

### 2.1. Screening of Lines for Resistance to F. oxysporum f. sp. Rapae

We have shown that *FocBr1* (Bra012688) is a resistance gene to *F. oxysporum* f. sp. *conglutinans* (*Foc*), and deletion of this gene results in susceptibility to *Foc* [12]; this susceptible allele is termed *focbr1-1* [14]. We made a DNA marker (Bra012688m) to detect the deletion of *FocBr1* that is homozygous for the *focbr1-1* allele [12,15]. In this study, we performed inoculation tests using *F. oxysporum* f. sp. *rapae* (*For*) for screening for resistant lines. Three Chinese cabbage (var. *pekinensis*), three turnip (var. *rapa*) and 22 komatsuna (var. *perviridis*) lines were tested of which 18 lines were resistant and 10 lines were susceptible (Table 1). We also inoculated these 28 lines with *Foc*, and resistance to *For* and *Foc* was identical (Table 1). We examined whether the results of the inoculation test were consistent with the prediction by *FocBr1* DNA marker (Bra012688m). In all lines of Chinese cabbage and turnip, the prediction by the DNA marker was identical to the resistance determined by the inoculation test, while in seven of 22 komatsuna lines (“Zaoh”, YBCG-12, YBCG-13, YBCG-14, YBCG-15, YBCG-TC02, and YBCG-TC05) the DNA marker prediction was not consistent with the results of the inoculation test (Table 1). We tested an additional 15 lines of *B. rapa*; three lines (“Chijimikomatsuna”, “Tsunashima”, and “Hirose”) were not consistent between the DNA marker prediction and the results of the inoculation test using *For* (Table S1).

### 2.2. Identification of the Causative Region of Resistance for F. oxysporum f. sp. Rapae

**Figure 1:**
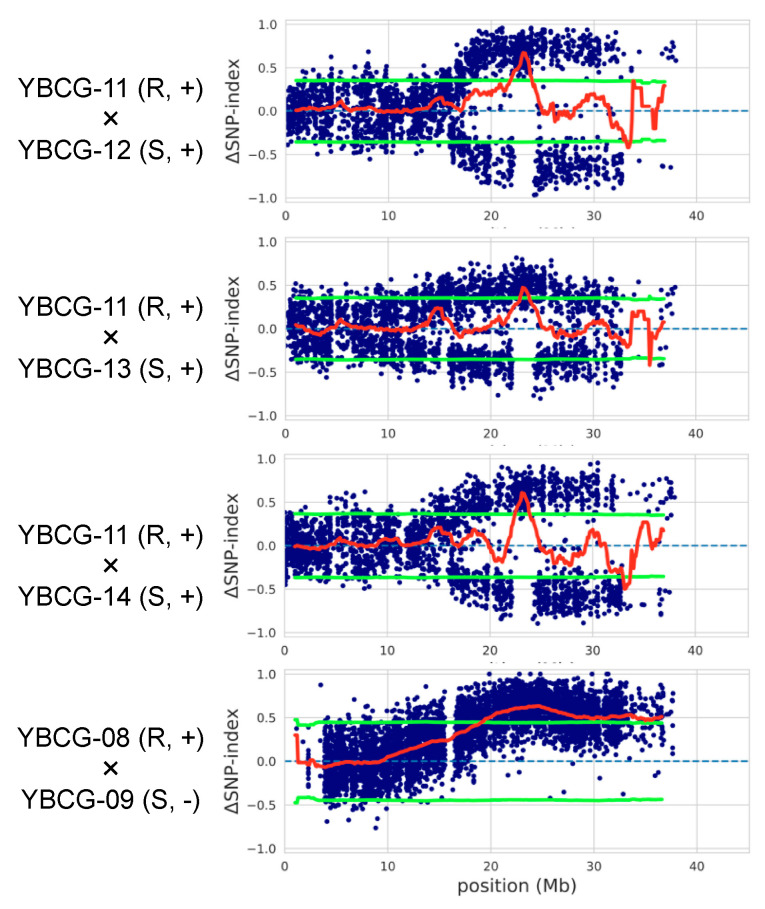
QTL-seq results on chromosome A03. F_2_ populations derived from YBCG-11 × YBCG-12, YBCG-11 × YBCG-13, YBCG-11 × YBCG-14, and YBCG-08 × YBCG-09 hybrids were used. Blue dots indicate ∆SNP-index, and the red line indicates the sliding window average of ∆SNP-index. Light green lines represent *p* < 0.05. R and S represent resistant and susceptible, respectively. + and - represent the presence and absence of PCR amplification of Bra012688m marker, respectively.

We performed linkage analysis using three individual F_2_ populations derived from hybrids between *For* susceptible lines not containing the *FocBr1* deletion and resistant lines. In the 200 plants of the F_2_ population derived from YBCG-11 (resistant) × YBCG-12 (susceptible) hybrid, 169 plants were resistant and 31 plants were susceptible to *For*. The number of susceptible plants was too small to be explained by a single gene dominance (chi-squared test, *p* < 0.05) (Table 2). This was also the case for the other two populations derived from YBCG-11 × YBCG-13 (susceptible) and YBCG-11 × YBCG-14 (susceptible) hybrids (Table 2). To identify the region covering the *R* gene for *For* (*ForBr1*), we performed QTL-seq analysis using bulked DNAs derived from about 20 resistant and susceptible individual plants derived from YBCG-11 × YBCG-12, YBCG-11 × YBCG-13, or YBCG-11 × YBCG-14 hybrids and found one similar locus on chromosome A03 in all three populations (Figure 1, Figures S1–S3). 22.0–33.5 Mb, 22.9–35.5 Mb, and 22.5–33.6 Mb region was detected as the QTL by 95% significance in the F_2_ population derived from YBCG-11 × YBCG-12, YBCG-11 × YBCG-13, and YBCG-11 × YBCG-14 hybrid, respectively (Figure 1). 1824, 1778, and 1734 genes were located in three QTLs, and 1655 genes overlapped (Figure S4, Table S2). A domain search using HMMSCAN with Pfam database and NCBI conserved domain search found nine genes encoding NBS-LRR proteins, including *FocBr1* (BraA03g047240.3C or Bra012688) (Table S3).

### 2.3. A New Susceptible Allele of FocBr1 Was Identified

**Figure 2:**
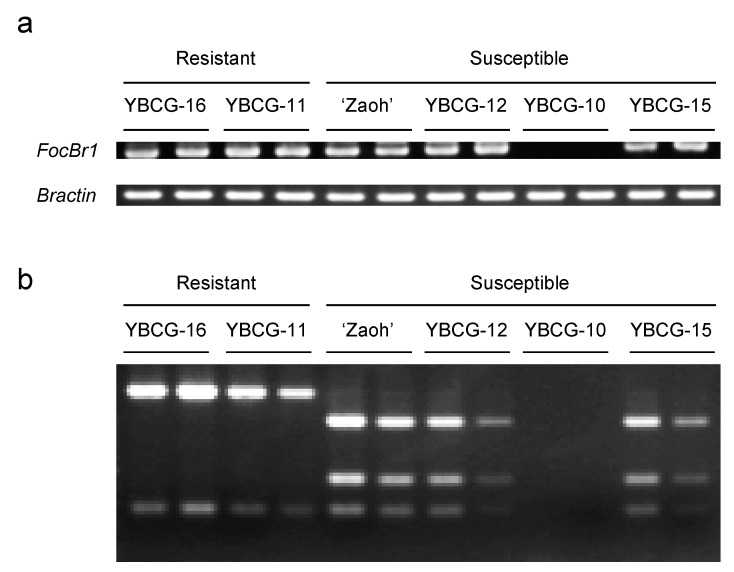
Expression and genotype of *FocBr1* in *For* resistant and susceptible lines. (**a**) Expression of *FocBr1* and *Bractin* (control) was confirmed by RT-PCR. (**b**) DNA fragments of RT-PCR products digested by *Hin*d III. YBCG-16 and YBCG-11 have *FocBr1*/*FocBr1* homozygous or *FocBr1*/*focbr1-1* heterozygous alleles. “Zaoh”, YBCG-12 and YBCG-15 have *focbr1-2*/*focbr1-2* homozygous or *focbr1-2*/*focbr1-1* heterozygous alleles, and YBCG-10 has *focbr1-1*/*focbr1-1* homozygous allele.

**Figure 3:**
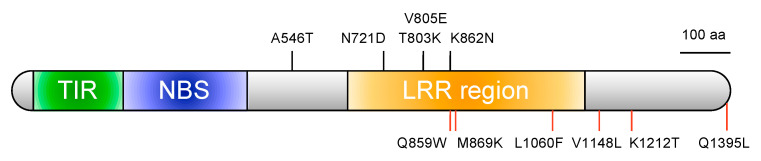
Protein structure of FocBr1 in the resistant line of *B. rapa*. TIR (green box), NBS (blue box), and LRR region (orange box) were identified. Black lines represent the position of difference of amino acid sequences between resistant and susceptible lines in *B. rapa*, while amino acid sequences of FocBr1 in the susceptible lines were identical to the FocBo1 (*Foc* resistance gene in *B. oleracea*). Red lines represent the position of susceptible line-specific amino acid substitutions. Domains were predicted using HMMSCAN with Pfam database. (https://www.ebi.ac.uk/Tools/hmmer/, accessed on 1 April 2021) and NCBI conserved domain search (https://www.ncbi.nlm.nih.gov/Structure/cdd/wrpsb.cgi, accessed on 1 April 2021).

Because *FocBr1* was included in three QTLs, we focused on *FocBr1* for further analysis. The expression level of *FocBr1* in three susceptible lines (“Zaoh”, YBCG-12, and YBCG-15) was similar to that of resistant lines (YBCG-11 and YBCG-16) (Figure 2), indicating that expression levels are not related to susceptibility. Next, we compared the amino acid sequences of FocBr1 in resistant and susceptible lines. The amino acid sequence of FocBr1 in the resistant line, YBCG-11, was 100% identical to FocBr1 in the resistant line, RJKB-T23 [12]. Amino acid sequences of FocBr1 were 100% identical among bulked DNAs of susceptible plants derived from F_2_ populations of YBCG-11 × YBCG-12, YBCG-11 × YBCG-13 and YBCG-11 × YBCG-14 hybrids, but there were some substitutions of amino acid sequences compared with FocBr1 in YBCG-11 (Figure 3). There were eleven amino acid sequence differences in FocBr1 between resistant and susceptible lines; five (A546T, N721D, T803K, V805E, and K862N) of which were identical between FocBr1 in the susceptible lines and the resistant allele of FocBo1 (*B. oleracea*) (Figure 3). Both FocBr1 and FocBo1 are resistance genes to *Foc*, indicating that the difference of amino acid sequences between FocBr1 and FocBo1 might not relate to the Fusarium yellows resistance, and the identical amino acid sequences between susceptible lines and FocBo1 might not lead to its susceptibility. The remaining six amino acid changes (Q859W, M869K, L1060F, V1148L, K1212T, and Q1395L) were differed between susceptible lines (YBCG-12, YBCG-13, and YBCG-14) and resistance lines of *B. rapa* and *B. oleracea* (Figure 3), which are susceptible line specific, suggesting that some of these amino acid sequences specific to susceptible lines result in susceptibility to *For*; some mutations may result in loss of function.

We examined whether *FocBr1* deletion (*focbr1-1*) causes susceptibility to *For*. Linkage analysis using three individual F_2_ populations derived from hybrids between *For* susceptible lines (*focbr1-1*) and resistant lines (YBCG-08 (resistant) × YBCG-09 (susceptible), YBCG-TC01 (resistant) × YBCG-10 (susceptible), and YBCG-11 × YBCG-10) showed that the number of resistant and susceptible plants segregated as 3:1 ratio (chi-squared test, *p* > 0.05) (Table 2). QTL-seq analysis using bulked DNAs derived from about 20 resistant and susceptible plants of the F_2_ population derived from YBCG-08 × YBCG-09 hybrid found one causative locus (19.0–36.6 Mb) on chromosome A03, which covers the *FocBr1* locus (Figure 1 and Figure S5). We tested a DNA marker (Bra012688m) in 12 resistant and susceptible plants from these three F_2_ populations, and the presence and absence of *FocBr1* was consistent with the inoculation test (Figure S6). These results indicate that *FocBr1* is essential for resistance to not only *Foc* but also *For*, supporting the suggestion that mutations cause susceptibility to *Foc* and *For*. This susceptible allele was termed *focbr1-2*. 

### 2.4. Development of a New DNA Marker for Fusarium Yellows Resistance

Using sequence polymorphism between *FocBr1* and *focbr1-2*, a new cleaved amplified polymorphic sequence (CAPS) DNA marker (focbr1-2m) was developed. Using this DNA marker, genotypes of 12 resistant and susceptible plants in three F_2_ populations derived from YBCG-11 × YBCG-12, YBCG-11 × YBCG-13, and YBCG-11 × YBCG-14 hybrids were confirmed, and genotypes were identical to the resistance determined by the inoculation test (Table S4). All ten lines that were not consistent between *For* inoculation test and the DNA marker (Bra012688m) prediction had homozygous *focbr1-2* or heterozygous *focbr1-2* and *focbr1-1* alleles (Table 1 and Table 3, and Table S1). Another 33 *B. rapa* lines were consistent between the inoculation test using *For* and prediction by DNA marker (focbr1-2m) (Table 3). These results indicate that this new DNA marker can detect not only the *focbr1-1* susceptible allele but also the *focbr1-2* allele.

### 2.5. Prediction of Fusarium Yellows Resistance in Commercial B. rapa Vegetables by DNA Marker

Using the focbr1-2m marker, we predicted the resistance to *For* in 157 cultivars of Chinese cabbage, 35 cultivars of turnip, 40 cultivars of pak choi, and 73 cultivars of komatsuna. Of 157 cultivars of Chinese cabbage, six cultivars (3.8%) were heterozygous for *FocBr1*/*focbr1-2*, and there were no cultivars homozygous for *focbr1-2*/*focbr1-2* or heterozygous for *focbr1-2*/*focbr1-1* (Table 4). There were six Chinese cabbage cultivars (3.8%) homozygous for *focbr1-1*/*focbr1-1* (Table 4), which could be susceptible to either *For* or *Foc*. Of 35 cultivars of turnip, five cultivars (14.3 %) were heterozygous for *FocBr1*/*focbr1-2*, and there were no cultivars homozygous for *focbr1-2*/*focbr1-2*, *focbr1-1*/*focbr1-1*, or heterozygous for *focbr1-2*/*focbr1-1* (Table 4). Of 40 cultivars of pak choi, 16 cultivars (40.0%) were heterozygous for *FocBr1*/*focbr1-2*, and there were no cultivars homozygous for *focbr1-2*/*focbr1-2*, *focbr1-1*/*focbr1-1*, or heterozygous for *focbr1-2*/*focbr1-1* (Table 4). Of 73 cultivars of komatsuna, 21 cultivars (28.8%) were heterozygous for *FocBr1*/*focbr1-2*, and five cultivars (6.8%) were homozygous for *focbr1-2*/*focbr1-2* or heterozygous for *focbr1-2*/*focbr1-1*. There were three cultivars (4.1%) homozygous for *focbr1-1*/*focbr1-1* (Table 4). Cultivars with *focbr1-2*/*focbr1-2* or *focbr1-2*/*focbr1-1* were found only in komatsuna.

## 3. Discussion

Previously, we identified a resistance gene to *Foc* (*FocBr1*), which is a single dominant gene. In susceptible lines, a 35 kb deletion, which includes *FocBr1,* was found [12], and the susceptible allele was termed *focbr1-1* [14]. A dominant DNA marker (Bra012688m) has been made and the prediction of *Foc* resistance using Bra012688m was consistent with phenotypes of *Foc* resistance confirmed by an inoculation test in inbred lines of Chinese cabbage [12,15]. In this study, we inoculated *Foc* and other formae speciales, *For*, to Chinese cabbage, turnip, pak choi, komatsuna, and chijimina lines, and we found the prediction using Bra012688m was not consistent with phenotype using inoculation test in some lines, especially in komatsuna. To clarify the inheritance pattern of the *R* gene to *For*, we performed linkage analysis. Three F_2_ populations derived from crosses between *For* resistant and susceptible lines (*focbr1-1*) showed a 3:1 ratio of resistant to susceptible plants. In contrast, the other three F_2_ populations derived from crosses between *For* resistant and susceptible lines (*focbr1-2*) did not show a 3:1 ratio of resistant to susceptible plants; the number of susceptible plants of the F_2_ population is smaller than the expected number. However, QTL-seq using these populations identified one causative locus. This could be a difference in the detail of the loss of function; FocBr1 of *focbr1-2* allele might have a weak function against *For* or be susceptible to environmental effects, although the *focbr1-1* allele has completely lost its function. However, as both alleles showed a strong susceptible phenotype and we could not identify any significant difference between these two alleles in *B. rapa* lines, further analysis will be needed to identify this minor difference between *focbr1-1* and *focbr1-2* alleles.

QTL-seq analysis using F_2_ populations derived from crosses between *For* resistant and susceptible lines with the *FocBr1* deletion (*focbr1-1*) or without the *FocBr1* deletion (*focbr1-2*) identified the same single causative locus for *For* resistance, which covered *FocBr1*. There was no difference in expression levels of *FocBr1* between resistant and susceptible lines, but there were some amino acid sequence differences between *For* susceptible allele *(focbr1-2*) and resistant alleles (*FocBr1* and *FocBo1*), suggesting that changes of amino acid sequence result in loss of function. Some substitutions were in the LRR region, and these susceptible line-specific amino acid changes may lead to loss of recognition of AVR. This new allele might be useful for identifying the sequence that is important for the interaction between R and AVR proteins. To prove this amino acid sequences change results in loss of function, which might be due to loss of recognition to AVR, further experiments such as making transgenic plants for complementation or loss of function by CRISPR-Cas9 system will be required.

Alternatively, another gene(s) linked to the *FocBr1* locus may work together with *FocBr1* for *For* resistance, because the peak detected by QTL-seq is upstream from the *FocBr1* position in three F_2_ populations derived from crosses between *For* resistant and susceptible lines (*focbr1-2*). In *Arabidopsis thaliana*, TIR-NBS-LRR type *Resistance to Ralstonia solanacearum 1* (*RRS1*) and *Resistance to Pseudomonas syringae 4* (*RPS4*) are neighboring genes and both are required for resistance to *Colletotrichum higginsianum*, *Ralstonia solanacearum,* and *Pseudomonas syringae* pv. *tomato* strain DC3000 expressing *avrRps4* [18]. *RRS1* encodes a WRKY domain protein as well as a TIR-NBS-LRR protein and works as a “sensor” to detect the effector, and RPS4 works as a “helper” to activate cell death [19,20]. If a similar function is applied to Fusarium yellows resistance, FocBr1 will work as a “sensor” NBS-LRR with other “helper” gene(s), which may be located on a region upstream from *FocBr1*. Resistant and susceptible alleles of “sensor”, *FocBr1* and *focbr1-2*, might be able to recognize AVR to greater or lesser degrees, respectively, and other “helper” gene(s) might have different functions between resistant and susceptible lines, resulting in a shift of QTL peak to upstream. Further analyses using plants recombined between QTL peak locus and *FocBr1* gene are required to clarify whether other factor(s) are important for *For* resistance and the *For* infection mechanisms. There is also another possibility that minor QTLs not linked to *FocBr1* locus are important for resistance to *For*.

Using a new DNA marker, focbr1-2m, we screened genotypes of *B. rapa* breeding lines and cultivars. There were six lines that showed weak resistance against *For* and *Foc*, and three of the six lines (“CR-Taiga”, “Natsurakuten”, and YBCG-TC04) showed heterozygosity of *FocBr1* and *focbr1-2*. However, in the remaining three lines (“CR-Yukiakari”, NSI-01, and YBCG-18), we cannot distinguish between the homozygosity of *FocBr1* and the heterozygosity of *FocBr1* and *focbr1-1*, because *focbr1-1* results in deletion of *FocBr1* and focbr1-2m cannot amplify *focbr1-1* allele. In *B. rapa*, plants having a homozygous clubroot resistance gene show more stable clubroot resistance than plants having a heterozygous resistance gene [21,22,23]. Three lines may be heterozygous for *FocBr1* and *focbr1-1*. In the case of *focbr1-1*, it is desirable to develop a DNA marker to distinguish between *FocBr1*/*focbr1-1* heterozygosity and *FocBr1*/*FocBr1* homozygosity, i.e., using a linked marker close to the 35-kb deletion [14]. In the case of *focbr1-2*, codominant DNA marker, focbr1-2m, can distinguish the heterozygosity of *FocBr1* and *focbr1-2* alleles, which will be useful for breeding stable Fusarium yellows resistant cultivars.

In Chinese cabbage cultivars, there were no cultivars homozygous for *focbr1-2*, and a few lines heterozygous for *FocBr1*/*focbr1-2*. In our previous study using the Bra012688m marker, there was complete agreement between the DNA marker-based prediction and the inoculation test in Chinese cabbage lines [15]. Thus, there is little risk that the presence of the *focbr1-2* allele leads to susceptibility during the breeding of Fusarium yellows resistant cultivars in Chinese cabbage. Like in Chinese cabbage, most turnip cultivars (about 85%) did not have *focbr1-2* alleles, so this allele will not be a problem for breeding. However, in pak choi, 40% of cultivars were heterozygous for *FocBr1* and *focbr1-2* alleles. In komatsuna, about 30% of cultivars were heterozygous for *FocBr1* and *focbr1-2* alleles and about 7% of cultivars were homozygous for *focbr1-2* allele or heterozygous for *focbr1-2* and *focbr1-1* alleles. For the breeding of Fusarium yellows resistant cultivars in pak choi or komatsuna, the presence of the *focbr1-2* allele should be mapped in breeding lines, and the DNA marker, focbr1-2m, developed in this study will be useful for DNA marker-assisted selection.

## 4. Materials and Methods

### 4.1. Plant Materials and DNA and RNA Extraction

The breeding lines and commercial F_1_ hybrid cultivars of *B. rapa* vegetables were used as plant materials (Table S5). F_2_ populations were produced by bud pollination of F_1_ hybrid crossing YBCG-11 × YBCG-12, YBCG-11 × YBCG-13, YBCG-11 × YBCG-14, YBCG-08 × YBCG-09, YBCG-TC01 × YBCG-10, and YBCG-11 × YBCG-10. Genomic DNA was isolated from leaves by the CTAB (cetyl trimethyl ammonium bromide) method [24]. Total RNA was isolated from noninoculated leaves of ten-days-old seedlings by the SV Total RNA Isolation System (Promega Co., Madison, WI, USA).

### 4.2. Inoculation Test

A strain of *F. oxysporum* f. sp. *rapae* (isolated from komatsuna) (provided by NARO, MAFF 240322) or *F. oxysporum* f. sp. *conglutinans* (isolated from cabbage) was used to prepare inocula. Liquid inocula were obtained by inoculating potato sucrose broth medium (200 g/L potato extract and 20 g/L sucrose in distilled water) with the isolate and shaking at 130 rpm on a rotary shaker for 1 week. Roots of ten-days-old seedlings were dipped in fungal spore suspension (fungal titer of ~5 × 10^6^) for 5 h and then transplanted into a cell tray filled with soil. Plants were grown in the greenhouse, and two or three weeks after inoculation, individual plants were scored for interaction phenotype (IP) based on six categories that are 0 (no symptoms in tops and roots), 3 (darkening of roots, slight top stunting, and no chlorosis), 5 (dark stunted roots, tops stunted, and slight chlorosis of cotyledons), 7 (severe stunting of roots and tops and strong chlorosis) and 9 (severe stunting, necrosis, and death). To show the phenotype of breeding lines and cultivars, the average IP among 25 seedlings were categorized into resistant (IP = 0) or susceptible (IP = 3–9). Average IP of most resistant lines/cultivars was around 0 and average IP of most susceptible lines/cultivars was 9. However, some of the 25 seedlings of the line showed IP = 0 while others showed IP ≥ 3, and these exceptions are represented by R*. In the linkage analysis, F_2_ seedlings were used for inoculation test, and phenotypes of individual seedlings were resistant (IP = 0) or susceptible (IP = 3–9). Chinese cabbage inbred lines RJKB-T23 and RJKB-T24 were used as a resistant and susceptible control [12].

### 4.3. QTL-Seq

QTL-seq was performed following the method described in [25]. From F_2_ populations derived from YBCG-11 × YBCG-12, YBCG-11 × YBCG-13, YBCG-11 × YBCG-14, and YBCG-08 × YBCG-09 hybrids, about 20 plants were selected from resistant (IP = 0) and susceptible (IP = 9) plants based on their perfectly resistance or susceptible phenotype. The equal amount of DNA from each sample was bulked by resistant and susceptible phenotypes, and named R-bulk and S-bulk, respectively. Eight sequence libraries were prepared for DNA sequencing using TruSeq DNA PCR-Free kit (Illumina, Inc., San Diego, CA, USA), and sequenced by Illumina Hiseq 4000 (paired end, 150 bp). For detecting the parental SNPs, DNA from parental line (YBCG-08) was also sequenced. 

Sequence reads were quality trimmed by FaQCs. Trimmed reads of R-bulk and S-bulk were aligned to the *B. rapa* reference genome version 3.0 (https://brassicadb.cn, accessed on 1 April 2021), and SNP-index was calculated at all SNPs in R-bulk and S-bulk compared with resistant parental sequences, then the subtracted value of SNP-index of R-bulk from SNP-index of S-bulk was calculated as ∆SNP-index using QTL-seq pipeline.

### 4.4. Prediction of Fusarium Yellows Resistance by DNA Markers

To predict the Fusarium yellows resistance, the dominant marker Bra012688m [15] and codominant CAPS marker, focbr1-2m, were used. PCR was performed using QuickTaq^®^HS DyeMix (TOYOBO Co., Ltd., Osaka, Japan). The reaction mixture was incubated in the thermal cycler (TaKaRa PCR Thermal Cycler Dice^®^ Gradient, Takara Bio Inc., Kusatsu, Japan) at 94 °C for 2 min following by 35 cycles of 94 °C for 30 s, 55 °C for 30 s, and 68 °C for 1 min. PCR products were detected by electrophoresis (i-MyRunII, COSMO BIO CO., LTD., Tokyo, Japan) using 1.0% agarose gel (Bra012688m). To distinguish the *FocBr1* and *focbr1-2* alleles, amplified DNA digested by *Hin*d III restriction enzyme were electrophoresed on 1.5% agarose gel. Two or more independent individual plants in each cultivar were tested for genotyping. The primer sets of the DNA markers are listed in Table S6.

### 4.5. Gene Expression Analysis

cDNA was synthesized from 500 ng total RNA using ReverTra Ace qPCR RT Master Mix with gDNA Remover (TOYOBO Co., Ltd.). The specificity of the primer set of *FocBr1* was first tested by electrophoresis of RT-PCR amplified products using QuickTaq^®^HS DyeMix on 1.5% agarose gel in which a single product was observed. RT-PCR conditions were 94 °C for 2 min followed by 35 cycles of 94 °C for 30 s, 55 °C for 30 s, and 68 °C for 30 s. The absence of genomic DNA contamination was confirmed by the PCR of the no RT control. To distinguish the *FocBr1* and *focbr1-2* alleles, amplified DNA by RT-PCR digested by *Hin*d III restriction enzyme were electrophoresed on 1.5% agarose gel. The primer sets for RT-PCR are listed in Table S6.

## 5. Conclusions

In this study, we identified *FocBr1* as a *For* resistance gene, and a new susceptible allele of *FocBr1*, *focbr1-2*, was identified in *B. rapa*. Furthermore, a new DNA marker, which can distinguish between *FocBr1*, *focbr1-1*, and *focbr1-2*, was developed. 

## Figures and Tables

**Table 1:** Assessment of Fusarium yellows resistance by inoculation test.

Name	Inoculation Test		Prediction by DNA Marker
	*For*	*Foc*	Bra012688m
**Chinese cabbage (var. *pekinensis*)**			
“W77”	R	R	+
RJKB-T23	R	R	+
RJKB-T24	S	S	-
**Turnip (var. *rapa*)**			
“CR-Yukiakari”	R *	R *	+
“Hekiju”	R	R	+
NSI-01	R *	R *	+
**Komatsuna (var. *perviridis*)**			
“CR-Taiga”	R *	R *	+
“Manaka”	R	R	+
“Nanami”	R	R	+
“Natsurakuten”	R *	R *	+
“Zaoh”	S	S	+
YBCG-08	R	R	+
YBCG-09	S	S	-
YBCG-10	S	S	-
YBCG-11	R	R	+
YBCG-12	S	S	+
YBCG-13	S	S	+
YBCG-14	S	S	+
YBCG-15	S	S	+
YBCG-16	R	R	+
YBCG-17	R	R	+
YBCG-18	R *	R *	+
YBCG-TC01	R	R	+
YBCG-TC02	S	S	+
YBCG-TC03	R	R	+
YBCG-TC04	R *	R *	+
YBCG-TC05	S	S	+
YBCG-TC06	R	R	+

R and S represent resistant and susceptible, respectively, to *Foc* or *For*. * represents weak resistance (some of the 25 seedlings showed IP = 0, while others showed IP ≥ 3). +, amplification by Bra012688m; -, no amplification by Bra012688m.

**Table 2:** Linkage analysis using six individual F_2_ populations.

	F_2_ Population		χ^2^ (R:S = 3:1)	Resistant Parent	Bra012688m	Susceptible Parent	Bra012688m
	Resistant	Susceptible					
1	169	31	*p* < 0.05	YBCG-11	+	YBCG-12	+
2	171	29	*p* < 0.001	YBCG-11	+	YBCG-13	+
3	171	29	*p* < 0.001	YBCG-11	+	YBCG-14	+
4	160	40	*p* > 0.05	YBCG-08	+	YBCG-09	-
5	149	51	*p* > 0.05	YBCG-TC01	+	YBCG-10	-
6	156	44	*p* > 0.05	YBCG-11	+	YBCG-10	-

+; amplification by Bra012688m, -; no amplification by Bra012688m.

**Table 3:** *F. oxysporum* f. sp. *rapae* resistance and *FocBr1* genotype determined by focbr1-2m marker.

Name	Inoculation Test	Prediction by DNA Markers	
	*For*	Bra012688m	focbr1-2m
**Chinese cabbage (var. *pekinensis*)**			
“W77”	R	+	A
RJKB-T23	R	+	A
RJKB-T24	S	-	D
RJKB-T36	R	+	A
RJKB-T37	R	+	A
RJKB-T38	R	+	A
RJKB-T39	R	+	A
RJKB-T40	S	-	D
**Turnip (var. *rapa*)**			
“CR-Yukiakari”	R *	+	A
“Hekiju”	R	+	A
“Yukibotan”	R	+	A
NSI-01	R *	+	A
**Pak choi (var. *chinensis*)**			
“Entei”	R	+	C
“Ryoutou”	R	+	A
**Komatsuna (var. *perviridis*)**			
“Chijimikomatsuna”	S	+	B
“CR-Taiga”	R *	+	C
“Kahoku”	R	+	C
“Manaka”	R	+	C
“Nakamachi”	R	+	A
“Nanami”	R	+	A
“Nanane”	R	+	A
“Natsurakuten”	R *	+	C
“Norichan”	R	+	A
“Tsunashima”	S	+	B
“Zaoh”	S	+	B
YBCG-08	R	+	A
YBCG-09	S	-	D
YBCG-10	S	-	D
YBCG-11	R	+	A
YBCG-12	S	+	B
YBCG-13	S	+	B
YBCG-14	S	+	B
YBCG-15	S	+	B
YBCG-16	R	+	A
YBCG-17	R	+	A
YBCG-18	R *	+	A
YBCG-TC01	R	+	A
YBCG-TC02	S	+	B
YBCG-TC03	R	+	A
YBCG-TC04	R *	+	C
YBCG-TC05	S	+	B
YBCG-TC06	R	+	A
**Chijimina (var. *narinosa*)**			
“Hirose”	S	+	B

R and S represent resistant and susceptible to *For*, respectively. * represents weak resistance (some of the 25 seedlings showed IP = 0, while others showed IP ≥ 3). +; amplification by Bra012688m, -; no amplification by Bra012688m. A, Resistant allele (*FocBr1*/*FocBr1* or *FocBr1*/*focbr1-1*). B, Susceptible allele (*focbr1-1*/*focbr1-2* or *focbr1-2*/*focbr1-2*). C, Heterozygous allele (*FocBr1*/*focbr1-2*). D, No PCR amplification (*focbr1-1*/*focbr1-1*).

**Table 4:** Genotype distribution of *FocBr1* in *B. rapa* subspecies.

	Chinese Cabbage	Turnip	Pak Choi	Komatsuna
	(var. *pekinensis*)	(var. *rapa*)	(var. *chinensis*)	(var. *perviridis*)
*FocBr1*/*FocBr1* or *FocBr1*/*focbr1-1*	145	30	24	44
*FocBr1*/*focbr1-2*	6	5	16	21
*focbr1-1*/*focbr1-1*	6	0	0	3
*focbr1-2*/*focbr1-2* or *focbr1-2/focbr1-1*	0	0	0	5
Total	157	35	40	73

## Supplementary Materials

The following are available online at https://www.mdpi.com/article/10.3390/plants10061082/s1, Figure S1: QTL-seq results of all chromosomes of F_2_ populations derived from YBCG-11 × YBCG-12 hybrid, Figure S2: QTL-seq results of all chromosomes of F_2_ populations derived from YBCG-11 × YBCG-13 hybrid, Figure S3: QTL-seq results of all chromosomes of F_2_ populations derived from YBCG-11 × YBCG-14 hybrid, Figure S4: Overlapped candidate genes in three F_2_ populations, Figure S5: QTL-seq results of all chromosomes of F_2_ populations derived from YBCG-08 × YBCG-09 hybrid, Table S1: Assessment of Fusarium yellows resistance by inoculation test, Table S2: Genes located on candidate locus detected by QTL-seq in four populations by 95% significance, Table S3: NBS-LRR encoded genes in significant QTLs, Table S4: Genotype using focbr1-2m marker in resistant and susceptible plants derived from F_2_ population, Table S5: List of cultivars used in this study, Table S6: List of primer sequences.
