# Supplementary material for: Development of a New DNA Marker for Fusarium Yellows Resistance in Brassica rapa Vegetables
Source: Plants (Basel). 2021 May 27;10(6):1082. doi: 10.3390/plants10061082 (PMC8229042; doi:10.3390/plants10061082)
Supplement: Supplementary file 1 [file plants-10-01082-s001.zip › Supplementary files/Supplemental Figures.pptx]

## Slide 1
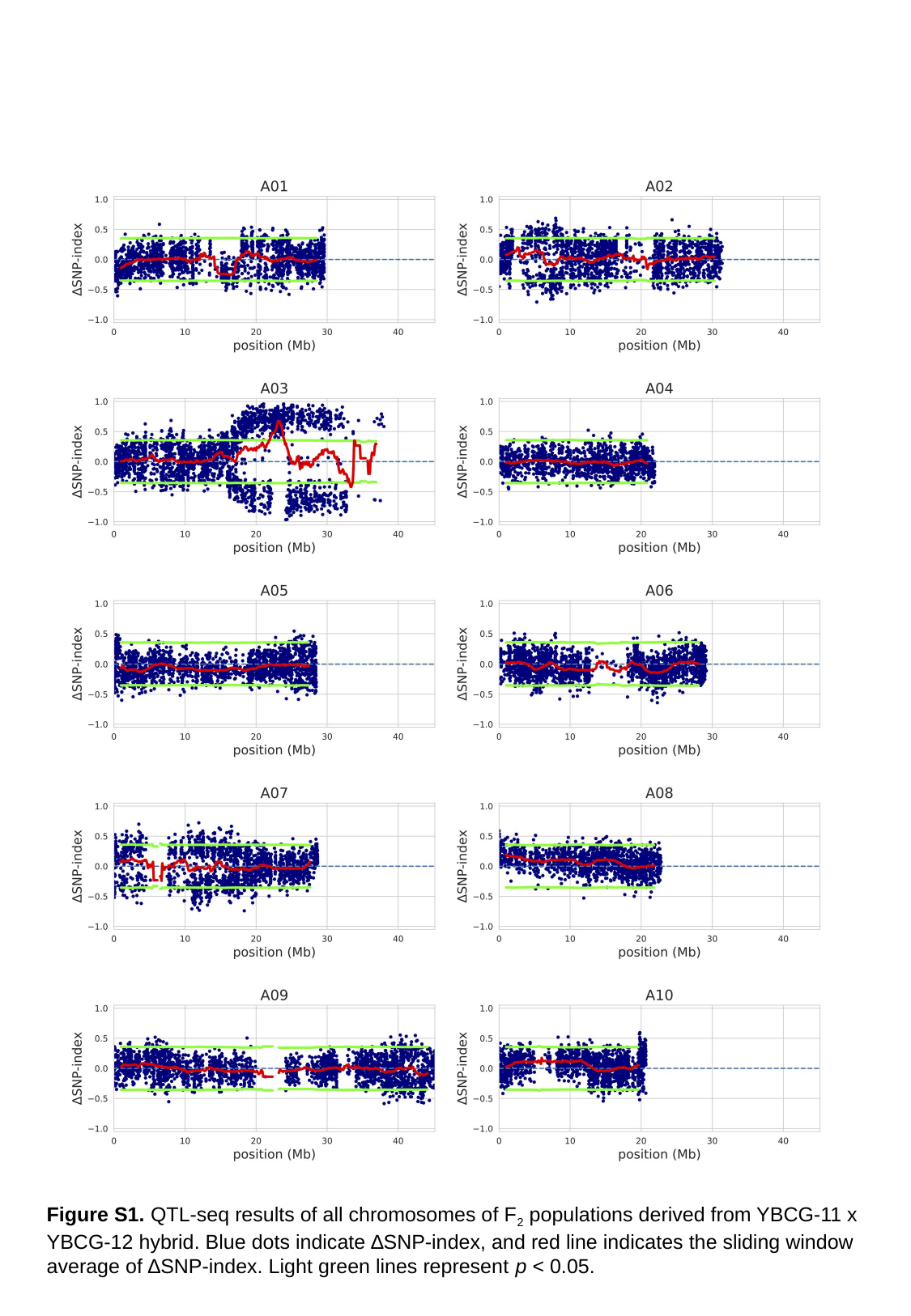

Figure S1. QTL-seq results of all chromosomes of F2 populations derived from YBCG-11 x YBCG-12 hybrid. Blue dots indicate ∆SNP-index, and red line indicates the sliding window average of ∆SNP-index. Light green lines represent p < 0.05.

## Slide 2
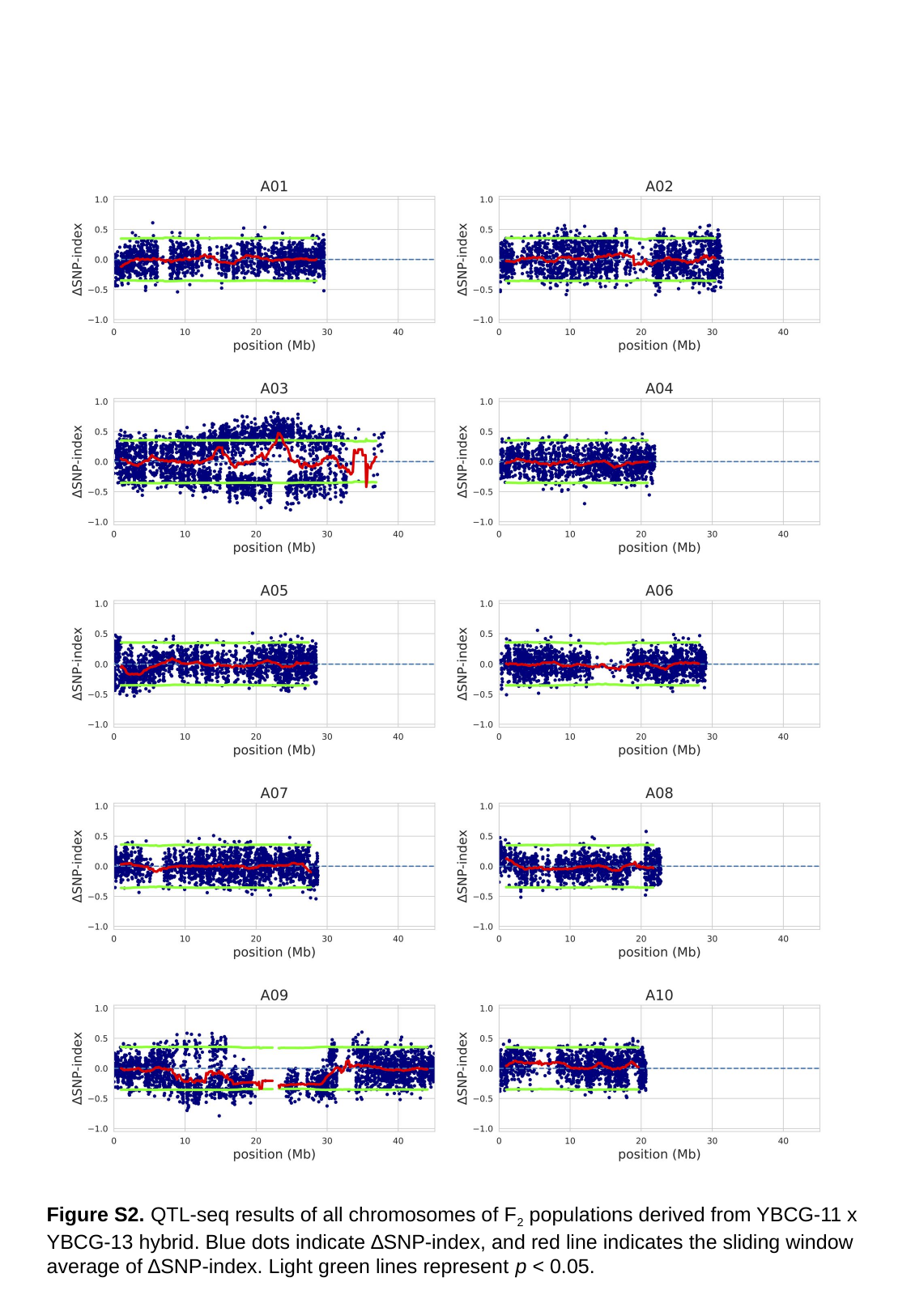

Figure S2. QTL-seq results of all chromosomes of F2 populations derived from YBCG-11 x YBCG-13 hybrid. Blue dots indicate ∆SNP-index, and red line indicates the sliding window average of ∆SNP-index. Light green lines represent p < 0.05.

## Slide 3
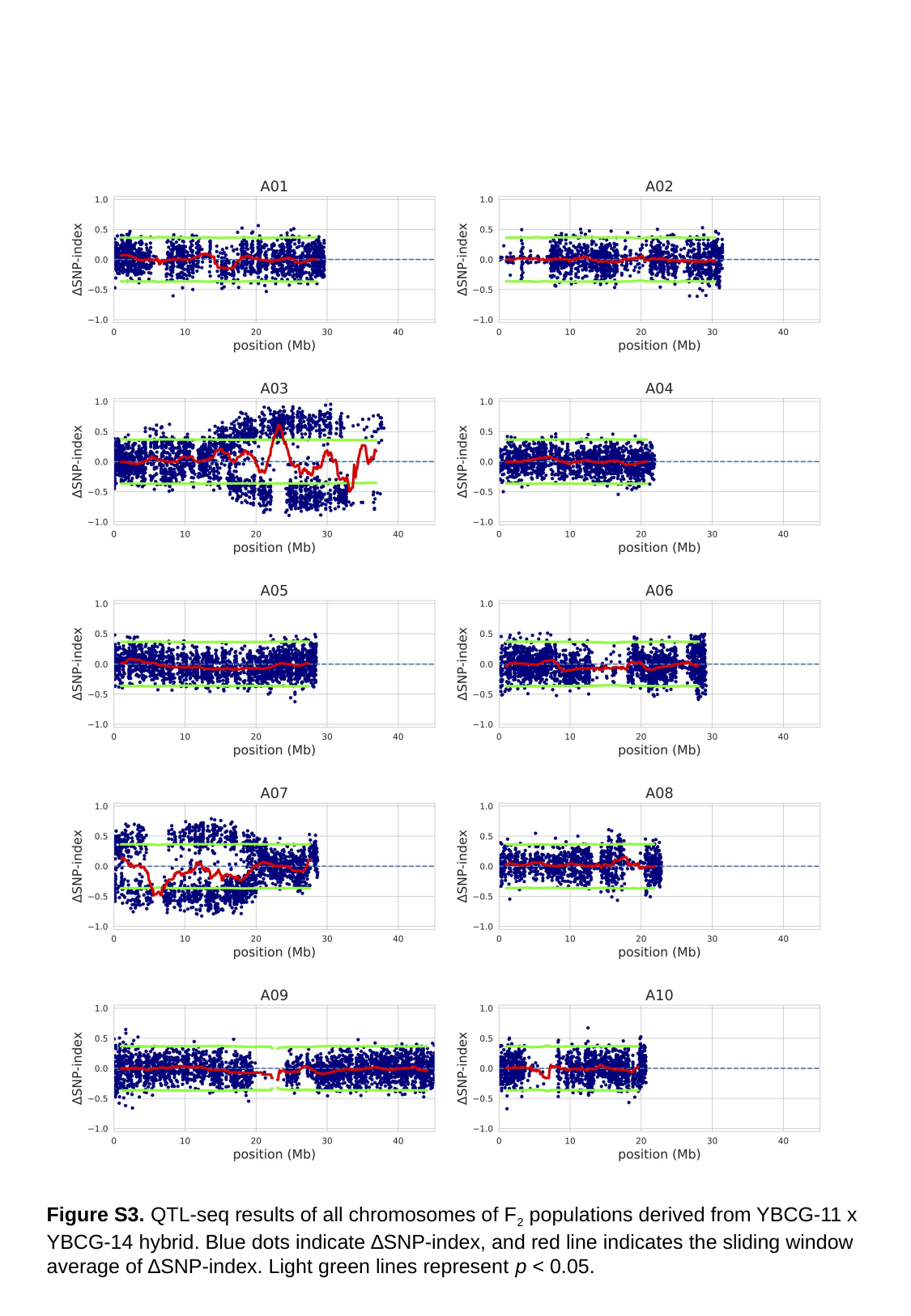

Figure S3. QTL-seq results of all chromosomes of F2 populations derived from YBCG-11 x YBCG-14 hybrid. Blue dots indicate ∆SNP-index, and red line indicates the sliding window average of ∆SNP-index. Light green lines represent p < 0.05.

## Slide 4
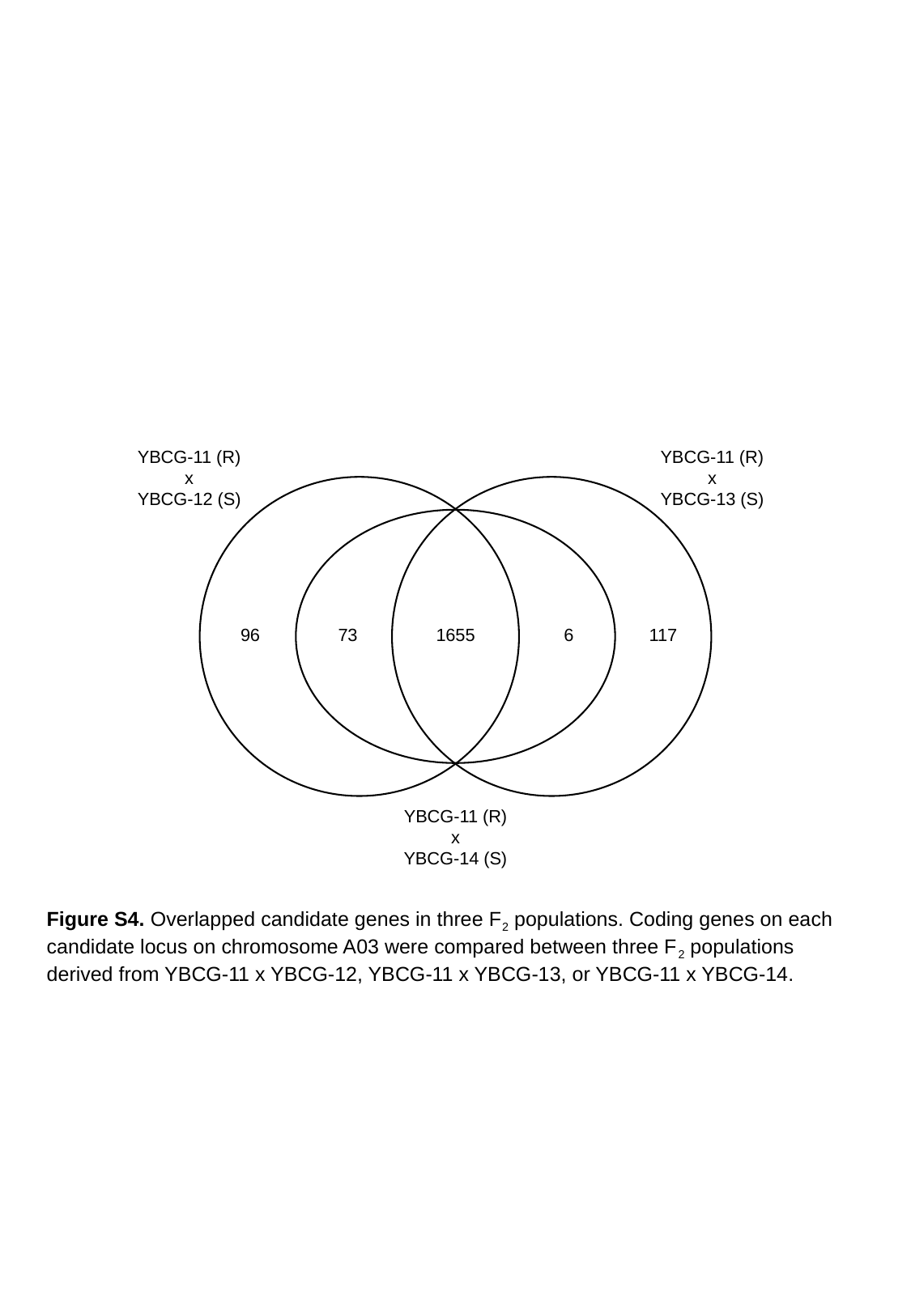

YBCG-11 (R)
x
YBCG-12 (S)
YBCG-11 (R)
x
YBCG-13 (S)
96
73
1655
6
117
YBCG-11 (R)
x
YBCG-14 (S)
Figure S4. Overlapped candidate genes in three F2 populations. Coding genes on each candidate locus on chromosome A03 were compared between three F2 populations derived from YBCG-11 x YBCG-12, YBCG-11 x YBCG-13, or YBCG-11 x YBCG-14.

## Slide 5
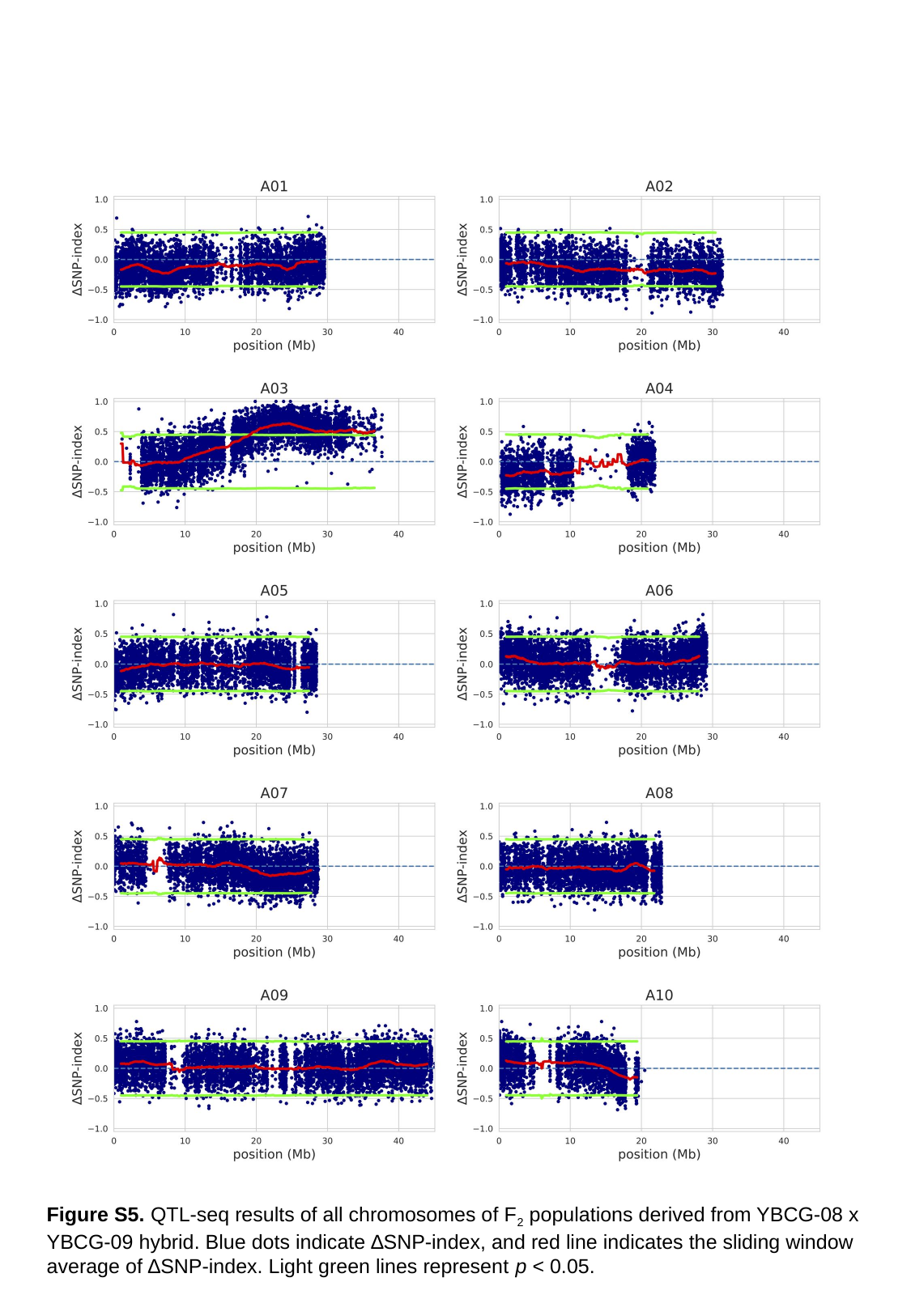

Figure S5. QTL-seq results of all chromosomes of F2 populations derived from YBCG-08 x YBCG-09 hybrid. Blue dots indicate ∆SNP-index, and red line indicates the sliding window average of ∆SNP-index. Light green lines represent p < 0.05.

## Slide 6
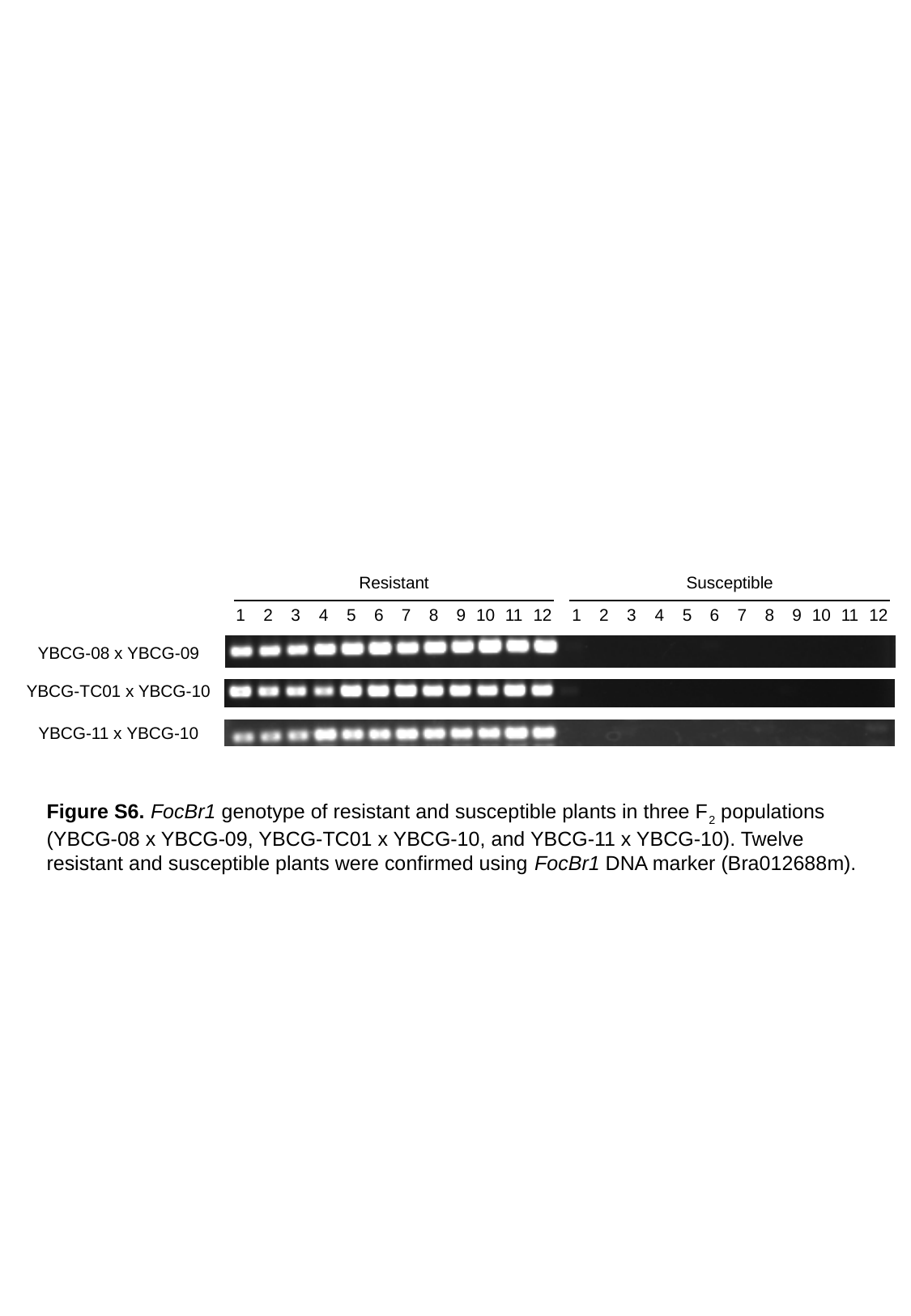

Resistant
1
2
3
4
5
6
7
8
9
10
11
12
Susceptible
1
2
3
4
5
6
7
8
9
10
11
12
YBCG-08 x YBCG-09
YBCG-TC01 x YBCG-10
YBCG-11 x YBCG-10
Figure S6. FocBr1 genotype of resistant and susceptible plants in three F2 populations (YBCG-08 x YBCG-09, YBCG-TC01 x YBCG-10, and YBCG-11 x YBCG-10). Twelve resistant and susceptible plants were confirmed using FocBr1 DNA marker (Bra012688m).
